# Roles of Toll-Like Receptors in Radiotherapy- and Chemotherapy-Induced Oral Mucositis: A Concise Review

**DOI:** 10.3389/fcimb.2022.831387

**Published:** 2022-06-02

**Authors:** Ling Ji, Siyuan Hao, Jiantao Wang, Jing Zou, Yan Wang

**Affiliations:** ^1^ State Key Laboratory of Oral Diseases, National Clinical Research Center for Oral Diseases, Department of Pediatric Dentistry, West China Hospital of Stomatology, Sichuan University, Chengdu, China; ^2^ State Key Laboratory of Biotherapy and Department of Lung Cancer Center and Department of Radiation Oncology, West China Hospital, Sichuan University, Chengdu, China

**Keywords:** toll-like receptor, oral mucositis, gastrointestinal mucositis, chemotherapy, radiotherapy, microbiota dysbiosis, oral microbiota

## Abstract

Radiotherapy and/or chemotherapy-induced oral mucositis (RIOM/CIOM) is a common complication in cancer patients, leading to negative clinical manifestations, reduced quality of life, and impacting compliance with anticancer treatment. The composition and metabolic function of the oral microbiome, as well as the innate immune response of the oral mucosa are severely altered during chemotherapy or radiotherapy, promoting the expression of inflammatory mediators by direct and indirect mechanisms. Commensal oral bacteria-mediated innate immune signaling *via* Toll-like receptors (TLRs) ambiguously shapes radiotherapy- and/or chemotherapy-induced oral damage. To date, there has been no comprehensive overview of the role of TLRs in RIOM/CIOM. This review aims to provide a narrative of the involvement of TLRs, including TLR2, TLR4, TLR5, and TLR9, in RIOM/CIOM, mainly by mediating the interaction between the host and microorganisms. As such, we suggest that these TLR signaling pathways are a novel mechanism of RIOM/CIOM with considerable potential for use in therapeutic interventions. More studies are needed in the future to investigate the role of different TLRs in RIOM/CIOM to provide a reference for the precise control of RIOM/CIOM.

## Introduction

Radiotherapy and/or chemotherapy-induced oral mucositis (RIOM/CIOM) is the most common localized oral complication in a large proportion of patients receiving radiotherapy and/or chemotherapy (Lalla et al., 2014). It seriously affects the quality of life of patients, increases the economic burden of treatment, and has a negative effect on antitumor treatment (Thomsen and Vitetta, 2018). Clinically, chemotherapy and radiotherapy are still two widely used, and effective approaches to treat a variety of cancers, including head and neck tumors (Siegel et al., 2019), aimed at improving survival (Bockel et al., 2018). Elting et al. reported that oral mucositis occurred in over 90% of the patients who received radiotherapy to head and neck primary cancers (Elting et al., 2007). According to Sonis, when the total cumulative dose of radiation in the mouth exceeds 30 Gy, the risk of oral mucositis is nearly 100% (Sonis, 2011). In patients with head and neck cancer receiving radiotherapy, the incidence rate of oral mucositis is up to 80% (Rubenstein et al., 2004). Over half of the patients who received altered fractionation radiation therapy and 34% of the patients who received conventional radiotherapy experienced severe mucositis (grades 3–4) (Trotti et al., 2003; Vera-Llonch et al., 2006; Wang et al., 2018). Even if the dose and frequency of chemotherapy and radiation were adjusted, the improvement and recovery were not satisfactory. Chemotherapy and radiation can also affect most of the alimentary canal. Patients developed mouth ulcers, erythema, pain, eating disorders, vomiting, and diarrhea complicated by weight loss and infectious diseases such as sepsis (Elting et al., 2003; Kusiak et al., 2020). These effects usually lead to more significant results, including malnutrition and prolonged hospitalization, and may be accompanied by chronic inflammation, necrosis, and systemic infection (Rosenthal and Trotti, 2009). The compliance of patients will critically decrease, contributing to breaks in radiotherapy (Saadeh, 2005) and a reduction in the dosage of chemotherapy drugs (Vera-Llonch et al., 2006). Moreover, these consequences have severely influenced patient compliance and quality of life (Gibson et al., 2015).

Growing evidence suggests that multidirectional interactions between the oral microbiota and the host innate immune system may influence the progression of inflammation caused by chemotherapy or radiotherapy (Cario, 2016). Radiotherapy and chemotherapy damage the epithelium of the oral mucosa, destroy the normal barrier structure (Thomsen and Vitetta, 2018), and contribute to oral microbiome dysbiosis, which further promotes the occurrence of mucositis (Vanlancker et al., 2018). Chemotherapy or radiotherapy can reduce the number and diversity of microbiota (Fijlstra et al., 2015). This change in microbiota composition allows rare microbial species to overgrow and shift the microbial community to disease-accelerating entities, which may promote aberrant innate immune signals in the oral mucosa during the development of oral mucositis (Touchefeu et al., 2014; Cario, 2016).

As a key receptor found intracellularly or on the surface of oral and gastrointestinal epithelial cells, which mediates the interaction between microorganisms and hosts, the role of TLRs in chemotherapy- or radiation-induced oral mucositis has been illuminated by an increasing number of studies (Sugawara et al., 2006; Uehara and Takada, 2008; Cario, 2016). Among these receptors, some can bind pathogenic bacteria, activate NF-κB, regulate downstream signaling pathways, and promote inflammatory cytokines, including interleukin-6 (IL-6) and tumor necrosis factor-alpha (TNF-α) (Khan et al., 2018). Some receptors are able to activate ATP-dependent transport pumps such as ATP-binding cassette subfamily B member 1/multidrug resistance P-glycoprotein (ABCB1/MDR1 P-gp) to expel harmful substances that are generated by radiotherapy and chemotherapy (Frank et al., 2015) or regulate the production of prostaglandin E2 (PGE2), granulocyte-macrophage colony-stimulating factor (GM-CSF), and platelet-activating factor (PAF), which promote the repair and regeneration of epithelial cells (Shi et al., 2019). Therefore, this review will summarize the current evidence concerning the potential involvement of the TLR signaling pathway and explore the potential of TLRs as a target for the treatment of RIOM/CIOM.

## Oral Mucositis

### Histological Changes and Disease Process

Various histological changes occur in RIOM/CIOM, including the decreased thickness of the oral mucosal epithelium and reduced density of basal cells (Sobue et al., 2018). Nearly all the cells and tissues of the oral mucosa, including the extracellular matrix, contribute to barrier injury (Sonis et al., 2004). Epithelial tight junctions are broken down to injure the oral mucosa and oral ulcers occur (Sonis, 2007). This process compromises the five phases proposed by Sonis (Sonis, 2004). In the first stage, radiation or chemotherapy drugs directly damage DNA double strands of the basal cells of the oral mucosa, causing the release of a large amount of reactive oxygen species and damaging the surrounding epithelium, tissues, and vascular endothelial cells (Sonis, 2009). These factors all lead to the second phase, in which reactive oxygen species, chemotherapy, and radiation therapy activate a variety of transcription factors, such as NF-κB, a powerful transcription factor that can activate genes related to tissue destruction. In the third phase, the signals are further amplified through positive feedback loops. Clinical symptoms occur in the fourth, ulceration phase, where the direct ulceration of mucosal tissues enriches for mostly gram-negative bacteria. This, in turn, activates macrophages to further amplify and exacerbate the inflammatory disease process. During the final healthy phase, the injury is contained, and the extracellular matrix sends out signals that promote cell proliferation and differentiation, and the normal oral microbial composition begins to rebuild, completing healing within four weeks (Sonis, 2010).

### Microbiota in the Pathogenesis of RIOM/CIOM

There is a mutually beneficial relationship between the microbial community and the host (Ye et al., 2013; Chang et al., 2021). For example, resident oral bacteria are able to prevent colonization by exogenous organisms, which have the potential to be pathogenic (Shu et al., 2020). Resident bacteria use a number of mechanisms to achieve this. They will compete with foreign organisms for available ligands and receptors for attachment. Because resident organisms are highly competitive with endogenous substrates in the oral cavity, exogenous organisms cannot thrive. The metabolic function of resident bacteria can create a variety of microenvironments, which usually produce conditions that are not suitable for other organisms to colonize, such as changes in pH or redox potential (Seminario-Amez et al., 2017). In addition, it has been shown that the coordinated regulation of host inflammatory responses between commensal oral bacteria and the host innate immune system is critical for the control and maintenance of healthy homeostasis (Chang et al., 2021). For instance, commensal oral bacteria can indirectly participate in innate defenses by modulating select defense mediators (Greer et al., 2013). Commensal oral bacteria can also indirectly participate in inflammation resolution to maintain coexistence with favorable microorganisms residing within the oral cavity through key homeostatic immunological regulators of host-commensal interactions in the oral mucosa (Nassar et al., 2017). By participating in nitrate metabolism, host-oral microbiome interactions play a significant role in the maintenance of cardiovascular health (Wade, 2013).

The oral microbial composition can remain stable for a long time in healthy individuals (Hu et al., 2013); and chemotherapy and radiotherapy have been shown to lead to significant changes in microbial composition that can contribute to the development of mucositis (Costello et al., 2009; Napenas et al., 2010; Thaiss et al., 2016; Sroussi et al., 2017). Chemotherapy and radiotherapy can disrupt the ecological balance in the oral cavity by damaging the oral epithelium and reducing the number of commensal microbes (Donnelly et al., 2003; Ye et al., 2013). Napenas et al. investigated 16S rRNA in different bacterial species before and after chemotherapy in nine breast cancer patients (Napenas et al., 2010). Of particular interest was the finding that 25 species (60%) were exclusive to the post-chemotherapy samples, which suggests a change in the oral microbiota following chemotherapy. A study of head and neck cancer patients receiving radiotherapy showed that *Candida* spp. were isolated in 77% of the patients with oral mucositis (Belazi et al., 2004). In another pediatric study, the presence of *Candida* spp. was shown to be associated with the increased severity of mucositis in children with acute lymphoblastic leukemia (de Mendonça et al., 2012). Lastly, Hou et al. studied the dynamics of the intraoral microbiota in patients treated with radiotherapy alone as well as radiotherapy in combination with chemotherapy and found that *Clostridium* spp., *Porphyromonas* spp., *Dense Helicobacter* spp., and *Puccinia* spp. showed dynamic and relatively synchronous changes in abundance in the bacterial community (Hou et al., 2018). These changes appeared to overlap with the temporal phase of severe mucositis episodes, suggesting that an increase in the clinical severity of oral mucositis is likely to be closely related to changes in the proportion of specific microorganism groups in the patient’s oral cavity. Hong et al. further elaborated that the severity of oral mucositis was related to the imbalance of oral microbiota groups, the enrichment of potential gram-negative pathogenic bacteria in severe mucositis, and a decrease in symbiotic bacteria associated with healthy tissues (Hong et al., 2019). Our previous research in mice also indicated that after receiving radiotherapy, the top 30 most abundant bacteria in the oral cavity changed significantly, with a significant increase in the total anaerobic bacteria (Wang et al., 2021; Zhu et al., 2021).

Chemotherapy and radiotherapy reduce microbial diversity, leading to an overpopulation of opportunistic pathogenic bacteria, disrupting the balance between the microbial community and host, leading to inflammatory responses in the oral mucosa (Vanhoecke et al., 2015; Ingrosso et al., 2021). Furthermore, the composition and metabolic function of oral microorganisms are significantly altered during chemotherapy (de Farias Gabriel et al., 2021). The binding of some oral microorganisms to pattern recognition receptors (PRRs), especially TLRs, activates the receptors and promotes the development of inflammation through a series of reactions that activate NF-ĸB (Vasconcelos et al., 2016). After recognition by TLRs, bacteria are processed and translocated into the cell, activating nucleotide-binding oligomerization domain-like receptors (NLRs). This, in turn, regulates downstream signaling and produces inflammatory factors, which further positively regulate the TLR-mediated inflammatory response (Bahri et al., 2010). Translocating commensal bacteria can even prolong the existence of already established ulcerations, impairing tissue healing (Vasconcelos et al., 2016).

**Figure 1:**
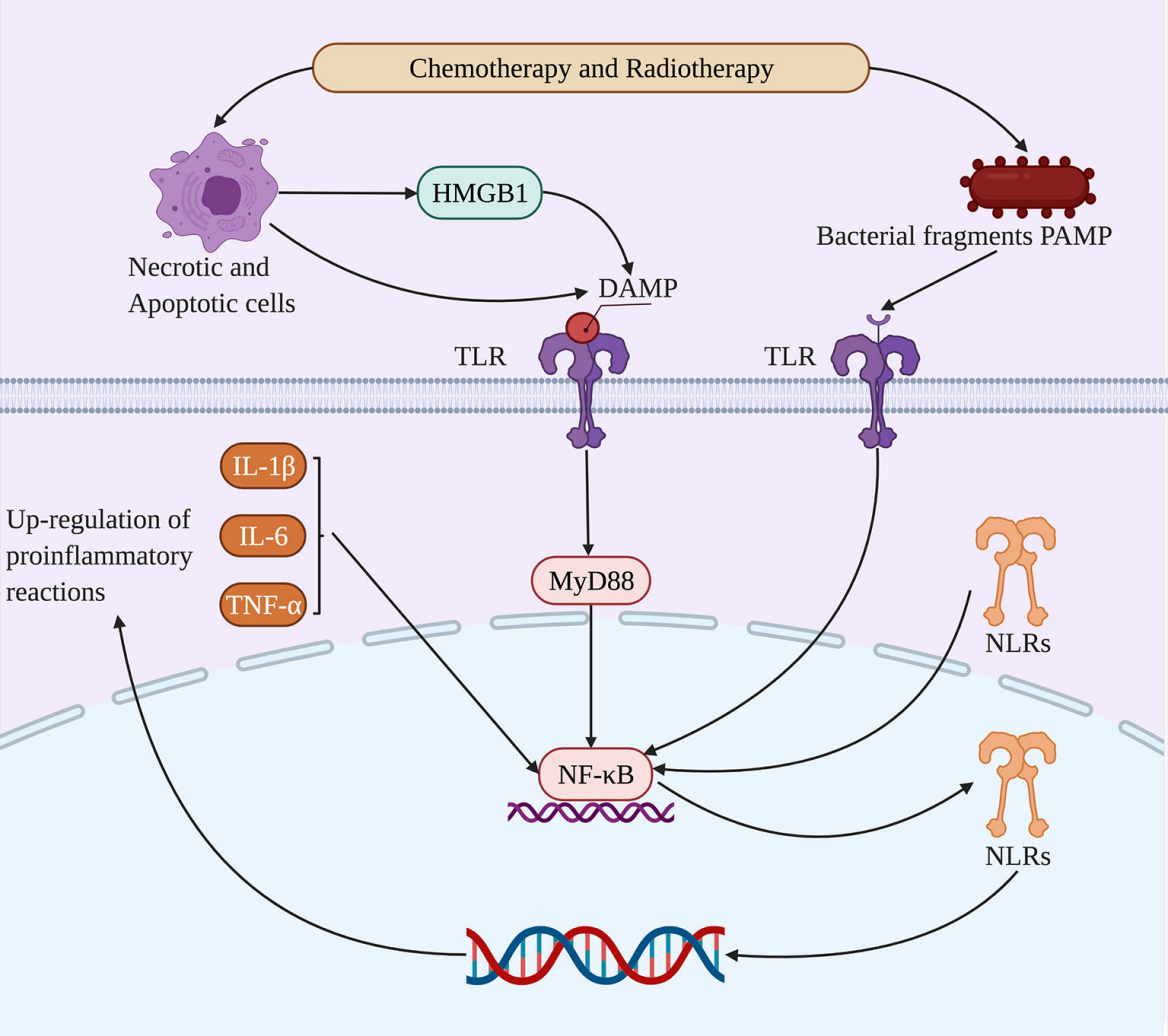
Microbial dysbiosis alters molecular pathways in RIOM/CIOM. Chemotherapy and radiotherapy lead to microbiota dysbiosis, after which TLRs can bind to HMGB1 as DAMPs from necrotic and apoptotic cells, as well as bacterial fragment PAMPs. Thus, TLRs upregulate NF-κB through the MyD88 signaling pathway and activate NLRs and proinflammatory reactions including IL-1β, IL-6, and TNF-α, promoting the damage of nucleic acids and the positive feedback of proinflammatory reactions to participate in RIOM/CIOM.

The occurrence of RIOM/CIOM not only contributes to microbial dysbiosis but also plays a role in the direct damage of epithelial cells, including DNA and RNA (Logan et al., 2008). The collection of pathogen-associated molecular patterns (PAMPS) and damage-associated molecular patterns (DAMPS), such as high mobility group protein box-1 (HMGB1), nucleotide fragments, and reactive oxygen species, disrupt normal gene expression and intensifies apoptosis by TLRs. This then activates a cascade of proinflammatory reactions to produce proinflammatory mediators such as IL-1β, IL-6, and TNF-α (Choi et al., 2018). Therefore, the involvement of the microbiome in the development of RIOM/CIOM, and PRRS such as TLRs are considered critical in its pathogenesis (Morales-Rojas et al., 2012; Wardill et al., 2016) (**Figure 1**).

## Toll-Like Receptors

TLRs are a family of molecules that are widely involved in mobilizing innate immunity, maintaining oral epithelial homeostasis, and signal transduction in the injury response stage of the oral mucosa (Groeger and Meyle, 2019). TLRs can respond to the endogenous molecular patterns caused by a variety of microorganisms and cell damage, recruit different junction proteins, and trigger a series of signaling cascade responses, resulting in effective defense mechanisms against invasive pathogens, tissue damage, or cancer, including the production of proinflammatory factors (Li et al., 2019). TLRs comprise a leucine-rich repeat (LRR) extracellular domain that can recognize ligands and a Toll/IL-1R (TIR) intracellular domain that is responsible for signal transduction (Mifsud et al., 2014). Current studies have shown that there are mainly myeloid differentiation factor (MyD88)-dependent pathways and MyD88-independent pathways in the signaling cascade amplification pathway (Hug et al., 2018), but TLRs can also activate extracellular regulated protein kinases (ERKs) and c-Jun N-terminal kinase (JNK) signal transduction upstream of mitogen-activated protein kinase (MAPK) and other signal transduction pathways (Anthoney et al., 2018).

Ten different kinds of TLRs have been discovered in humans, and their specific ligands have also been identified (Kawai and Akira, 2007). TLR1, TLR2, TLR4, TLR5, and TLR6 are mainly expressed on the cell membrane. According to their ligands, TLRs can be divided into three categories: TLR1, TLR2, TLR4, and TLR6, which bind lipid species; TLR5, which recognize pathogen proteins; and TLR3, TLR7, TLR8, and TLR9, which bind nucleic acids including DNA and RNA from both cells and viruses (Rauta et al., 2014). The typical microbial components shared by bacteria can stimulate the immune-inflammatory cascade in healthy oral mucosal epithelial cells, thereby maintaining the balance between normal oral microbial bacteria and the host (Sigal, 2005). However, excessive, deregulated inflammation can disrupt the normal function of healthy subgingival plaque biofilms with the concomitant disruption of its functional properties in relation to innate defense surveillance and tissue maintenance, leading to tissue destruction (Darveau and Curtis, 2021). This is the functional basis for the pattern recognition of TLRs (Takeda and Akira, 2004).

At present, all ten TLR mRNAs can be detected in oral epithelial cells (Groeger and Meyle, 2019). Beklen et al. obtained tissue samples from patients and confirmed that TLR1 to TLR9 were differentially expressed in the oral mucosal epithelium in addition to TLR10 using immunohistochemical techniques to localize TLRs in tissue samples (Beklen et al., 2008). Except for TLR7 and TLR8, all TLRs showed statistically significant differences after periodontal inflammation and normal tissue control. After obtaining oral mucosal epithelial tissue samples, Sugawara et al. found that Toll-like receptors were expressed in normal oral mucosal epithelium, especially TLR4 and TLR2 by immunohistochemical analysis (Sugawara et al., 2006). They also demonstrated that primary cultured oral epithelial cells expressed TLR4 and TLR2 by PCR, flow cytometry, and immunostaining; and its cell surface location was more pronounced in inflamed oral epithelial tissues than in healthy tissues (Lukova et al., 2020).

**Figure 2:**
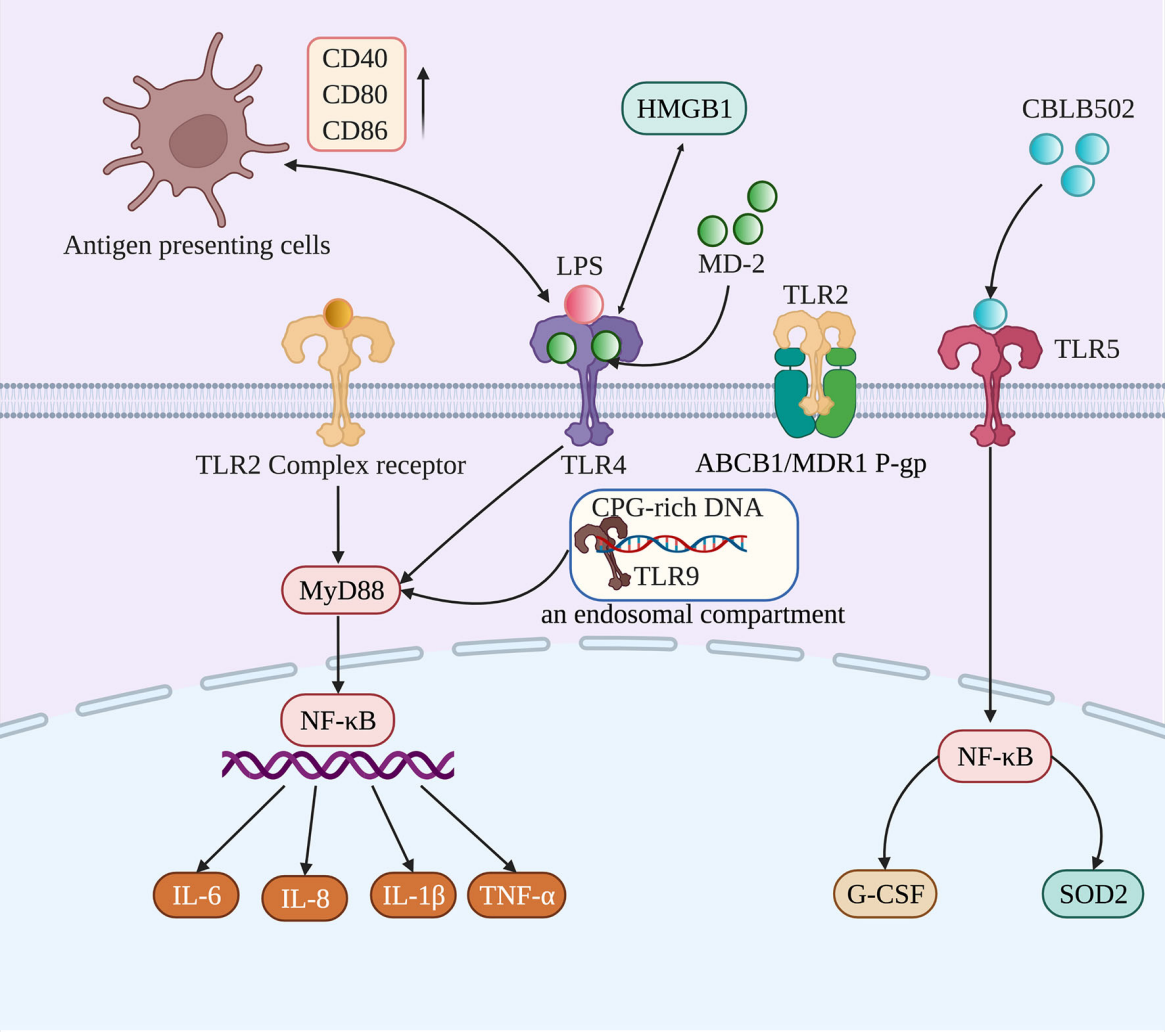
TLR2, TLR4, TLR5, and TLR9 participate in the occurrence and development of RIOM/CIOM through different mechanisms. Activated TLR2, TLR4, and TLR9 induce the expression of proinflammatory cytokines (IL-6, IL-8, IL-1β, and TNF-α) to participate in the development of RIOM/CIOM through the MyD88/NF-κB signaling pathway. TLR2 can be activated by forming a functional complex with other TLRs, and TLR2 can also activate ABCB1/MDR1 P-gp to prevent harmful substances from accumulating in cells. Due to chemotherapy and radiotherapy, TLR4 could not only bind to HMGB1 and myeloid differentiation protein 2 (MD-2), and be activated through interactions with LPS, but could also interact with antigen-presenting cells, accompanied by the upregulation of costimulatory molecules, such as CD40, CD80, and CD86. When activated by CBLB502, TLR5 induces protective cytokines (G-CSF) and antioxidants (SOD2), contributing to rebuilding the epithelium. As for TLR9, it can be activated after recognizing CpG DNA from bacteria.

The expression of TLRs plays an important role in maintaining the homeostasis of the oral epithelium. The most likely relevant defense mechanism mediated by TLR signaling in the oral cavity is the induction of antimicrobial substances, such as defensins (α-type, β-type, and θ-type), and the expression of human β-defensin (hBD)-1 to hBD-4 mRNA and protein in the oral epithelium (Liu et al., 2014). The mechanisms regulating TLR signaling in oral mucosal epithelial cells include the differential expression of TLRs and their signaling partners. Its cellular localization and mode in oral mucosal tissues affect its response to pathogens while attenuating the response to commensals and maintaining homeostasis under physiological tissue conditions (McClure and Massari, 2014). In this, human periodontal ligament cells (hPDLFs) can play an important role in the immune response of the periodontal microenvironment by secreting proinflammatory cytokines. Lipopolysaccharide (LPS) is a component of gram-negative bacteria and is a potent stimulator of TLR4 (Brown et al., 2010). A previous study identified the unique LPS-sensing mechanism of the oral epithelium, whereby the activation of TLR4 in gingival epithelial cells required vesicular acidification to turn on TLR4 signaling, indicating the stringency for fine-tuning a local LPS response (Kantrong et al., 2019). It has been confirmed that the mRNA and protein levels of TLR increased after the initial LPS stimulation but were found to be reduced after the secondary challenge. This process favors hPDLFs in maintaining oral mucosal immune homeostasis (Wu et al., 2015). After fungal, bacterial, and viral pathogens bind to TLRs, the downstream signaling pathways are activated and play an important role in innate and adaptive immune responses. Numerous microorganisms are constantly present in the oral mucosa, so the expression and function of TLRs are necessary to maintain oral mucosal homeostasis (Groeger and Meyle, 2019). The following details the specific roles of various TLRs in RIOM/CIOM **(** **Figure 2** **).**

## TLR2 and RIOM/CIOM

At present, the activation of TLR2 is generally believed to increase the expression of proinflammatory cytokines and participate in signaling pathways important in the main injury response period of oral mucositis induced by radiotherapy and chemotherapy (Villa and Sonis, 2016). TLR2 is highly expressed in the basal layer of the gingival epithelium and plays an important role in tissue homeostasis (Beklen et al., 2008). For example, TLR2 can recognize pathogens in the basal layer of the mucosal epithelium to promote the occurrence of TLR-dependent inflammation (Groeger and Meyle, 2019). Moreover, TLR2 can form a functional complex with other TLRs and activate NF-κB through the MyD88 signaling pathway. Studies have shown that the expression of NF-κB in human oral mucosa increased after chemotherapy (Frings et al., 2016), which may be related to the occurrence of oral mucositis after chemotherapy. NF-κB is a key downstream transcription factor activated through the TLR signaling pathway. After activation, it is transferred to the nucleus to bind to the target gene promoter region, and transcription induces the production of many inflammatory factors, such as IL-1β, IL-6, and IL-8 (Stringer and Logan, 2015). In contrast, TLR2 may function in protecting the mucous membrane. TLR2 has been shown to protect the intestinal mucosal barrier by activating ABCB1/MDR1 P-glycoprotein (ABCB1/MDR1 P-gp) (Frank et al., 2015). ABCB1/MDR1 P-gp is an ATP-dependent efflux transport pump that prevents the accumulation of foreign bodies in cells, thereby reducing the production of harmful substances in cells during chemotherapy (Cario, 2016).

Cario *et al.* used TLR2-/- mice, MyD88-/- mice, and wild-type mice to establish *in vitro and in vivo* models of intestinal epithelial cells to assess spontaneous apoptosis by terminal deoxynucleotidyl transferase-mediated dUTP-biotin nick end-labeling (Cario et al., 2007). The resulting data showed that TLR2 could control mucosal inflammation by modulating epithelial barrier function. Moreover, in intestinal mucosal inflammation, synthetic Pam3Cys-SK4 (PCSK), a ligand for TLR2, significantly inhibited mucosal inflammation and apoptosis by restoring the integrity of intestinal epithelial tight junctions *in vivo*. To date, there are relatively few studies on TLR2 and RIOM/CIOM. More related studies are needed to explain the relationship between TLR2 and oral mucositis (**Figure 2**).

## TLR4 and RIOM/CIOM

Although, the results of existing studies focusing on the role of TLR4 seem to be controversial, the activation of TLR4 is widely believed to increase the expression of several proinflammatory cytokines, including IL-6 and TNF-α (Kawai and Akira, 2010). Some studies have found that TLR4 was closely related to the occurrence and development of some inflammatory oral diseases, such as recurrent aphthous ulcer and periodontitis. For example, Karasneh et al. found a significant association between the TLR4 rs10759931 polymorphism and recurrent aphthous stomatitis, suggesting that TLR4 may be a therapeutic target for the treatment of oral ulcers (Karasneh et al., 2015). Moreover, Qi et al. conducted controlled experiments in a mouse model of periodontitis that showed periodontal inflammation upregulated TLR4 levels, enhanced cellular immunity, and affected endogenous transcription factor expression, thereby increasing the inflammatory response *in vivo* (Qi et al., 2019). However, the inhibition or knockdown of TLR4 receptors in mice effectively reduced periodontal inflammatory responses and cellular immunity. This suggests that TLR4 may be relevant as a therapeutic target for the inflammation of periodontal tissues in the oral cavity. Together, these results have some implications for oral mucositis. DAMPs, such as HMGB1, bind to TLR4 and activate TLR4 by interacting with LPS due to chemotherapy and radiotherapy. The inflammatory cascade reaction is then initiated. The TLR4 signaling pathway can stimulate host cells to produce proinflammatory cytokines such as TNF-α (Stringer and Logan, 2015), resulting in ulcers after damaging the basal layer of the mucosal epithelium, which runs through the entire oral epithelium (Villa and Sonis, 2016). In addition, the interaction between TLR4 and antigen-presenting cells can lead to the upregulation of costimulatory molecules, such as CD40, CD80, and CD86, on antigen-presenting cells, which may aggravate mucosal epithelial inflammation (Karasneh et al., 2015). Hamada et al. reported that in the intestinal mucosa of methotrexate (MTX)-treated rats, a significant increase in TLR4 mRNA and protein expression was observed and coincided with a proinflammatory setting (Hamada et al., 2013). TLR4 deficiency resulted in significantly reduced acute NF-κB signaling, inflammation, and COX-2 expression in a preclinical model of dextran sulfate sodium (DSS)-induced colitis (Fukata et al., 2005).

Several studies also showed that TLR4 signaling played a protective role. One study showed a protective mechanism in oral epithelial cells, where TLR4 was internalized and could not be activated by the major virulence factor LPS to prevent a hyper response to oral commensal bacteria (Kantrong et al., 2019). Both TLR4 and Myd88 protein and mRNA levels were significantly down-regulated in MTX-treated rats compared to control animals due to a compensatory mechanism in which activated receptors mediated their own downregulation to limit or stop the response to the stimulus (Sukhotnik et al., 2014). When activated by bacterial LPS, TLR4 has been shown to provide protection to the intestinal epithelium from radiation damage, increasing crypt survival and prostaglandin activity (Egan et al., 2004). According to previous clinical studies, severe chemotherapy-induced gastrointestinal mucositis was prevented in TLR4 gene knockout mice. However, TLR4 knockout mice completely lack an IL-6 response, and IL-6 may be a more promising therapeutic target for preventing or alleviating chemotherapy-induced gastrointestinal mucositis (Khan et al., 2018). In this way, it plays a protective role in gastrointestinal mucositis. Moreover, in DSS-induced or radiation-induced colitis and injury models, Shi et al. reported that mice with TLR4 deficiency were more susceptible to DSS-induced and radiation-induced intestinal damage, although they did not produce as many proinflammatory cytokines as their wild-type counterparts (Shi et al., 2019). In addition, the moderate activation of TLR4 by LPS promoted the expression of PGE2 and GM-CSF, repairing the epithelium in DSS and radiation-induced damage (Shi et al., 2019). Based on the above results, we believe that the role of TLR4 is to maintain a balance between protection and damage. When it is over-activated, its proinflammatory effect dominates, leading to the further aggravation of mucositis. However, if TLR4 is knocked out or inhibited completely, it will not be able to activate innate immunity and promote epithelial repair. Therefore, maintaining a balance in TLR4 activation is crucial in treating RIOM/CIOM **(** **Figure 2** **).**

## TLR5 and RIOM/CIOM

It has been demonstrated that the activation of TLR5 could ameliorate RIOM/CIOM. When the same agonist was tested in a mouse model it not only reduced the extent of oral mucosa damage but also accelerated regeneration (Toshkov et al., 2017). CBLB502, a TLR5 agonist, was administered to mice under single-dose and fraction local radiation of the head and neck and significantly alleviated mucositis (Burdelya et al., 2012). Burdelya et al. found that CBLB502 could be used as a ligand and agonist of TLR5, which mainly mediates the activation of downstream signaling pathways through TLR5 signaling pathways in normal tissue or tumors (Burdelya et al., 2012). The mice in the experimental group were subcutaneously injected with different doses of CBLB502 30 min before radiotherapy. The observation and comparison of the bodyweight of the mice and the pathomorphology of the mice mucous membranes between the experimental group and the control group, showed that CBLB502 could significantly reduce the severity of oral mucositis and accelerate the recovery of mucosal tissue. Moreover, under some radiation doses, CBLB502 reduced the degree of radiation-induced weight loss in mice after a single dose of radiotherapy. This study suggests that CBLB502 has the characteristics of both supportive therapy (radiotherapy adjuvant) and anticancer drugs. The mechanism was that TLR5 activated NF-κB signaling, which further induced antioxidant-designated superoxide dismutase 2 (SOD2) and radioprotective cytokine granulocyte colony-stimulating factor (G-CSF) (Burdelya et al., 2008). By suppressing the oxidative stress of ROS and contributing to restoration, they effectively reduced radiation toxicity (Atkinson et al., 1995). Furthermore, the activation of TLR5 had no protective effect on locally irradiated syngeneic head and neck mouse tumors (Burdelya et al., 2008). Attributed to the immunostimulatory effect of TLR5 signaling activation, tumor necrosis increased, leading to significant tumor regression (Rhee et al., 2008). In this case, a TLR5 agonist may be an ideal agent for treating RIOM/CIOM **(** **Figure 2** **).**

## TLR9 and RIOM/CIOM

The role of TLR9 in RIOM/CIOM is not well documented. TLR9 is a receptor that recognizes PAMPs such as unmethylated cytosine-phosphate-guanine motif (CpG DNA) from bacteria, and plays an important role in innate and adaptive immunity (Varga et al., 2016; Dragasevic et al., 2018). TLR9 has been confirmed to play an inflammatory and destructive role in the gastrointestinal tract (Donnelly et al., 2014). After chemotherapy, TLR9 is more likely to identify a part of the intestinal microbiota, send downstream signals, produce proinflammatory mediators, and mediate the occurrence of gastrointestinal mucositis. Adriamycin is an antibiotic used in anti-tumor therapy that easily induces intestinal injury during clinical treatment. The TLR9 antagonist ODN2088 can block TLR9 signal transduction and significantly reduce adriamycin-induced intestinal injury (Dragasevic et al., 2018). TLR9 antagonists can reduce intestinal injury caused by antineoplastic drugs. Some studies have also found that TLR9 receptor gene deletion could improve the survival rate of animals and reduce intestinal injury and bacteremia. At the same time, it decreased the expression of inflammatory markers, such as NF-κB, IL-1, IL-18, and COX-2 (Ribeiro et al., 2016). Wong et al. also illuminated that, compared to wild-type mice that were injected with saline or irinotecan to induce intestinal mucositis, TLR9 gene knockout preserved mucosa architecture, bacterial translocation, and the expression of IL-1β (Wong et al., 2015). However, the improving trend in diarrhea and survival did not achieve statistical significance, which may have been due to the different chemotherapy agents administered. Therefore, the current evidence demonstrates that activated TLR9 plays a proinflammatory role in mucositis, suggesting that the inhibition of TLR9 may also be a potential therapeutic target for RIOM/CIOM. However, more work is still needed to clarify the role of TLR9 in RIOM/CIOM (**Figure 2**).

## Conclusion

**Figure 3:**
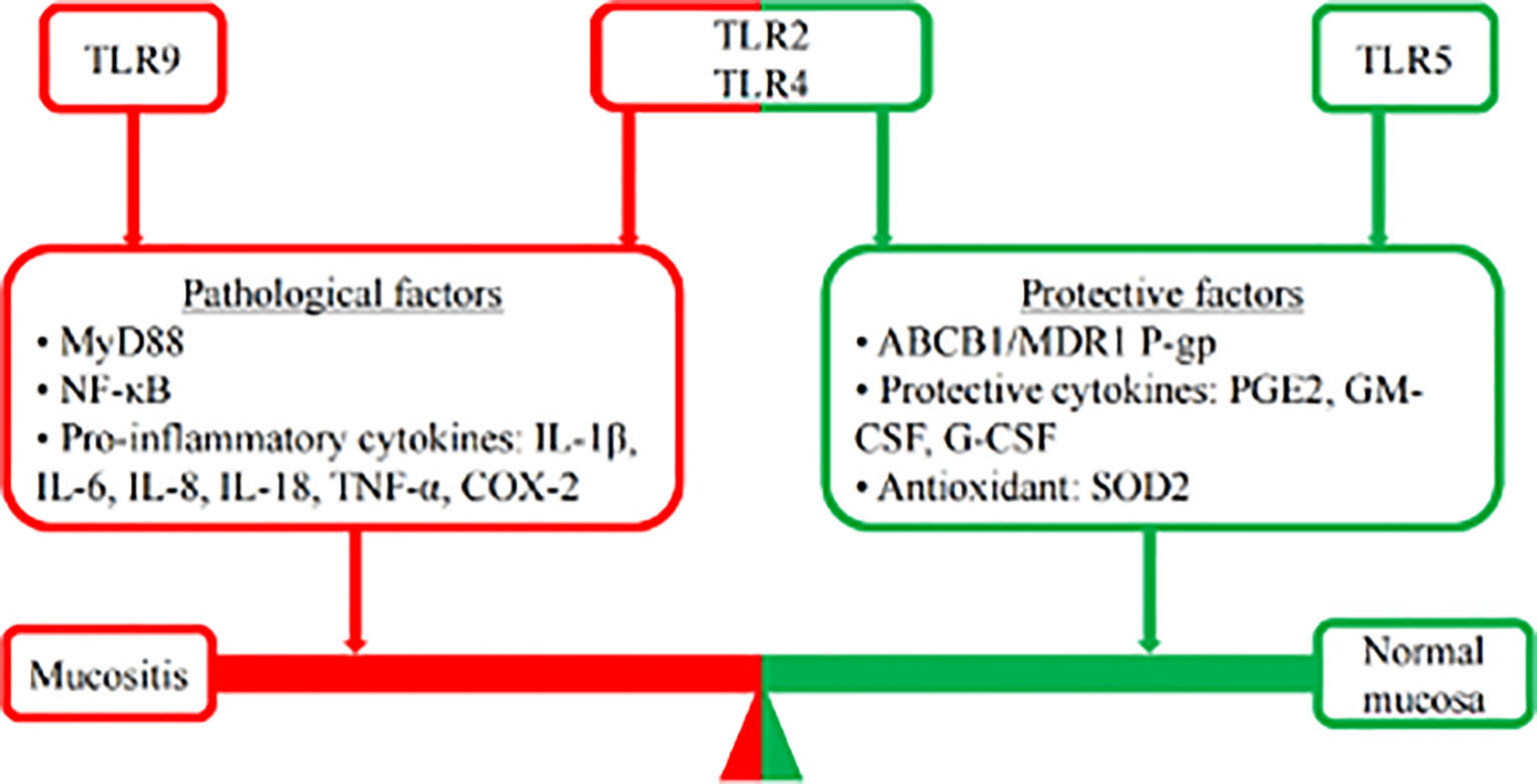
Mucosis balance. The mucositis process is recognized as a balance between pathological factors and protective factors. Following chemotherapy, TLR9 recognizes the microbiota and increases the expression of proinflammatory cytokines, such as NF-κB, IL-1, IL-18, and COX-2, to promote the development of mucositis. In contrast, TLR5 could suppress the oxidative stress of ROS to contribute to the restoration of RIOM/CIOM by inducing SOD2 and G-CSF. The role of TLR2 and TLR4 seems to be controversial. TLR2 and TLR4 could not only upregulate NF-κB to contribute to the increase in pathological factors (IL-1β, IL-6, IL-8, TNF-α, *etc.*) through the MyD88 signaling pathway, promoting RIOM/CIOM but also inducing ABCB1/MDR1 P-gp and protective cytokines (PGE2, GM-CSF, G-CSF, *etc.*) to promote the restoration of normal mucosa.

With the continuous deepening and accumulation of research, the role of TLRs in RIOM/CIOM is becoming clearer. The mucositis process is recognized as a balance between pathological factors and protective factors. TLR9 recognizes the microbiota and increases the expression of proinflammatory cytokines. In contrast, TLR5 could suppress the oxidative stress of ROS to contribute to the restoration of RIOM/CIOM by inducing SOD2 and G-CSF. TLR2 and TLR4 could not only upregulate NF-κB to contribute to the increase in pathological factors through the MyD88 signaling pathway, promoting RIOM/CIOM but also induce ABCB1/MDR1 P-gp and protective cytokines to promote the restoration of normal mucosa (**Figure 3**). The development of agonists and antagonists is also a promising direction for the clinical treatment of RIOM/CIOM in the foreseeable future. Of the studies reviewed, evidence suggests that the inhibition of TLR2 and TLR5, and the activation of TLR9 may be plausible in the protection of RIOM/CIOM. The role of TLR4 may need to be cautiously balanced to exert its therapeutic effect. Further studies in this area are expected to reveal the role of TLRs and their interaction with the microbiome (**Figure 3**).

## Author Contributions

LJ and SH drafted the manuscript. YW, JW, and JZ edited and added valuable insights to the manuscript. All authors contributed to the article and approved the submitted version.

## Funding

This study was supported by the National Natural Science Foundation of China (81600864 to YW) and the Undergraduate Student Innovation and Entrepreneurship Training Program of Sichuan University (C202114146 to SH). None of the funders played a role in the writing of the manuscript.

## Conflict of Interest

The authors declare that the research was conducted in the absence of any commercial or financial relationships that could be construed as a potential conflict of interest.

## Publisher’s Note

All claims expressed in this article are solely those of the authors and do not necessarily represent those of their affiliated organizations, or those of the publisher, the editors and the reviewers. Any product that may be evaluated in this article, or claim that may be made by its manufacturer, is not guaranteed or endorsed by the publisher.
